# A Global View of DNA-Packing Proteins Cracks the Histone Code

**DOI:** 10.1371/journal.pbio.0030346

**Published:** 2005-08-30

In one of biology's most impressive engineering feats, specialized proteins package some six-and-a-half feet of human DNA into a nucleus that averages just 5 microns (0.0001969 inches) in diameter. In the first of a series of supercondensing steps, DNA winds around proteins called histones, which together form a complex called the nucleosome. Histones package DNA into repetitive coils, which not only provide genomic structure but also help regulate gene expression. These tasks are mediated in part by chemical modifications to histone proteins—most commonly to histone “tails,” long, unstructured chains of amino acids that protrude from nucleosomes. Different chemical modifications are associated with different functional effects. Acetylation, which adds an acetyl group to an amino acid on the histone tail, has been linked to both gene activation and silencing, depending on which amino acid is modified. Methylation (addition of a methyl group to the histone tail) has also been linked to gene activation and repression, although the chemical effects of methylation differ dramatically from those of acetylation.

Even in yeast, amino acid modifications in the histone tails can number in the tens and twenties. Given the number of possible permutations of modification types and amino acids, the question arose, might different combinations of histone modifications produce discrete outcomes? The notion that a sequence or combination of specific modifications on histone tails acts as a signal to other proteins and produces distinct biological effects was advanced as the “histone code” hypothesis in 2000.[Fig pbio-0030346-g001]

**Figure pbio-0030346-g001:**
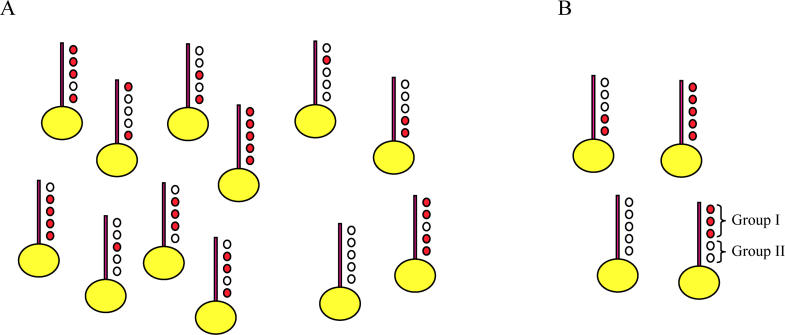
Histones can undergo many potential modifications, and it has been hypothesized that these can occur in many different combinatorial histone modification patterns (A). In this study, researchers found that only a few modification patterns occur in yeast, with many of the modifications co-occurring in groups (B)

Progress in deciphering the vocabulary, mechanics, and function of the histone code has been hindered by the coarse resolution of available tools. Nucleosomes typically cover about 146 base pairs, but existing technology could only average over 500 to 1,000 base pairs at a time—confounding the effects of single nucleosomes. In a new study, Oliver Rando and colleagues take advantage of the high resolution afforded by their custom-made microarray, which has a resolution of 20 base pairs. Working with the budding yeast *Saccharomyces cerevisiae*, the scientists examined 12 different histone modifications in individual nucleosomes and found only a small number of distinct combinations with “few discrete histone modification patterns.” The concurrent modifications fall into two categories: one set targets a transcriptional start site but is the same no matter what the level of transcription, while the other occurs throughout gene coding regions and is linked to transcription. Importantly, the only modifications that appear to correlate with transcription occur over transcribed regions, as though they were the consequence, rather than the cause, of transcription.

Why might histone tails exhibit so many modifications if they form only two independent categories? It's possible that histone-modifying enzymes may work best in groups and so the marks that recruit them—acetyl and methyl groups—also come in groups. Another possible explanation relates to how histone modifications signal transcription enzymes that a particular gene requires more or less transcription. When the positively charged amino acid lysine acquires an acetyl group, it loses its charge, and charge–charge interactions play a major role in many interactions between proteins and other molecules. Multiple lysine acetylations on the histone tail may thereby aid certain chemical reactions necessary for transcription in a continuous way; having multiple levels of acetylation, for example, may allow the cell to “tune” protein–protein interactions, and thus gene expression, up and down, rather than simply turn it on or off.

Rando and colleagues propose that the histone modifications associated with transcription may facilitate rather than trigger gene expression, perhaps by clearing a path for the transcription machinery or attracting proteins needed for the job. The authors are careful to point out, however, that histone modifications may also play some role in initiating gene expression, but that any transcription pattern would likely be obscured, or “erased,” as transcription occurs. While future studies will help determine which role proves more common, these results suggest that histone modifications are facilitators rather than activators and that the histone code is more a transcription footprint than a starting signal. —*Liza Gross*

